# Region specific Raman spectroscopy analysis of the femoral head reveals that trabecular bone is unlikely to contribute to non-traumatic osteonecrosis

**DOI:** 10.1038/s41598-017-00162-3

**Published:** 2017-03-07

**Authors:** Tristan Pascart, Guillaume Falgayrac, Henri Migaud, Jean-François Quinchon, Laurène Norberciak, Jean-François Budzik, Julien Paccou, Anne Cotten, Guillaume Penel, Bernard Cortet

**Affiliations:** 1 Lille University, Littoral Côte d’Opale University, EA 4490, PMOI, Physiopathologie des Maladies Osseuses Inflammatoires, F-59000 Lille, France; 2 Department of Rheumatology, Saint-Philibert Hospital, Lille University, F-59160 Lomme, France; 3 Department of Orthopaedic Surgery, Lille University Hospital, Lille University, F-59000 Lille, France; 4 Department of Anatomopathology, Saint-Philibert Hospital, Lille University, F-59160 Lomme, France; 5 Department of biostatistics, Saint-Philibert Hospital, Lille University, F-59160 Lomme, France; 6 Department of Radiology,Saint-Philibert Hospital, Lille University, F-59160 Lomme, France; 7 Department of Rheumatology, Lille University Hospital, Lille University, F-59000 Lille, France; 8 Department of Radiology, Lille University Hospital, Lille University, F-59000 Lille, France

## Abstract

Non-traumatic osteonecrosis (ON) of the femoral head is a common disease affecting a young population as the peak age of diagnosis is in the 40 s. The natural history of non-traumatic ON leads to a collapse of the femoral head requiring prosthetic replacement in a 60% of cases. Although trabecular bone involvement in the collapse is suspected, the underlying modifications induced at a molecular level have not been explored in humans. Here, we examine changes in the molecular composition and structure of bone as evaluated by Raman spectroscopy in human end-stage ON. Comparing samples from femoral heads harvested from 11 patients and 11 cadaveric controls, we show that the mineral and organic chemical composition of trabecular bone in ON is not modified apart from age-related differences. We also show that the molecular composition in the necrotic part of the femoral head is not different from the composition of the remaining ‘healthy’ trabecular bone of the femoral head. These findings support that quality of trabecular bone is not modified during ON despite extensive bone marrow necrosis and osteocyte death observed even in the ‘healthy’ zones on histological examination.

## Introduction

Non-traumatic osteonecrosis (ON) of the femoral head is a common disease with an estimated annual incidence of 3/100,000 in Europe^1^ and up to 29/100,000 in Asia^2^. The disease affects a young population as the peak age of diagnosis is in the 40 s^3^. The natural history of ON leads to a collapse of the femoral head requiring prosthetic replacement in a 60% of cases^3–5^.

Research on the pathophysiology of the disease has not identified a single mechanism and a multiple hit theory has been proposed including vascular occlusion, direct cellular toxicity, altered mesenchymal stem cell differentiation, dysfunctional lipid metabolism and anatomical abnormalities^4, 6–8^. How the induced infarction eventually leads to the collapse of the femoral head is however not fully understood. Cancellous bone has been suspected to be responsible for the defective mechanical properties of the osteonecrotic femoral head^9^. This suspicion led to explorations of trabecular bone quality during the course of ON using different techniques applied at various scales. The macroscale was investigated with dual-energy X-ray absorptiometry. The bone mineral density (BMD) of osteonecrotic femoral heads was found to be decreased compared with matched controls^10, 11^. At the mesoscale, histological analyses showed necrosis and decreased osteocyte viability beyond the necrotic zone and in some cases as far as the proximal femur^12–15^. Micro-computed tomography was applied to study the microscale^16^. Wang *et al.* found local alterations of the microarchitecture in 10 necrotic femoral heads. These authors reported cracks and thinning of trabeculae in the necrotic region but a normal microstructure at a distance^17^. The molecular scale remains to be explored in depth, particularly looking for local discrepancies, to provide a better understanding of bone quality alterations in affected bone.

Raman spectroscopy enables simultaneous exploration of mineral and organic composition and structure in healthy and pathological bone^18^. Physicochemical parameters (PCPs) can be measured to assess relative variations in the composition and structure of a bone sample, providing a reliable grasp of its quality at a molecular level. Raman spectroscopy is increasingly used to understand how changes in bone composition and structure influence tissue-level mechanical properties of bone^19–22^. Raman spectroscopy can be performed on fresh samples using simple sample preparation to the contrary of other vibrational techniques analyzing bone composition such as Fourier transform infrared (FTIR)^23, 24^. Raman spectroscopy is thus a promising tool to explore the molecular changes occurring in bone during ON. Aruwajoye *et al.* used Raman spectroscopy to examine early modifications in an animal model of ON of the femoral head and found increased carbonate substitution in the necrotic bone^25^. Animal models provide insight into early-stage ON, but cannot fully reproduce the features of human disease; such results must be confronted with analyses of human samples^26^.

So far, the mechanisms leading to the collapse of the femoral head and the anatomical extent of the altered bone remain unclear such that the structural evolution is still unpredictable^4^. The objective of this study was to examine modifications of the molecular composition and structure of bone as evaluated by Raman spectroscopy in human end-stage non-traumatic ON of the feoral head, and to search for relations with histological findings.

## Results

### Raman Spectroscopy

**Figure 1:**
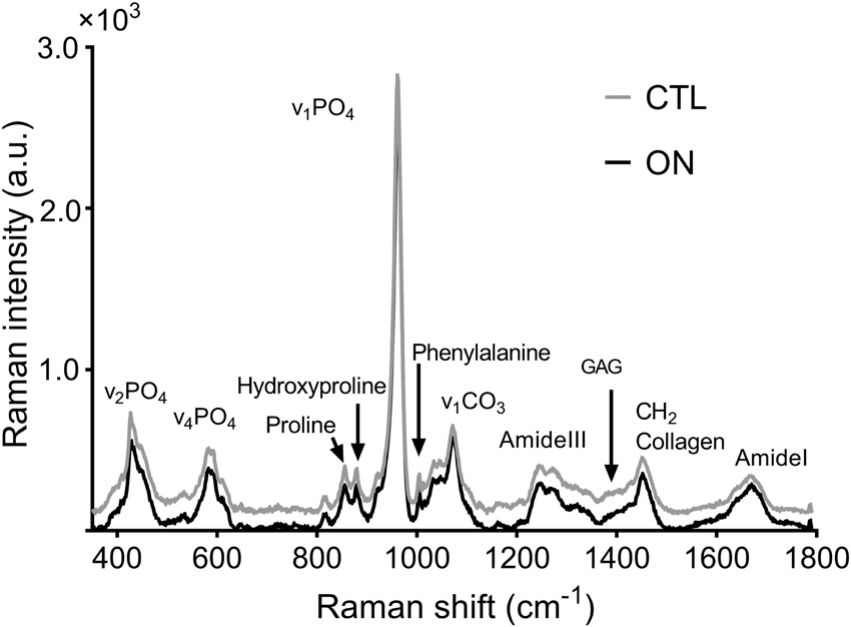
Mean Raman spectra representative of the necrotic zone in osteonecrosis (ON) group and of the necrotic equivalent zone in the control (CTL) group.

The Raman spectrum of bone is shown in Fig. 1. Values of each PCP according to the zones of sampling are presented in Table 1.

**Table 1:** Mean values ± Standard Deviation (Standard Error) for each physicochemical parameter for the necrotic, sclerotic and distant zones of the non-traumatic osteonecrosis (ON) of the femoral head group and equivalent zones of the control group.

Group	Zone	Proteoglycan relative content	Collagen maturity	Hydroxyproline-to-proline ratio	Mineral-to-matrix ratio	Carbonate-B-substitution	Crystallinity
ON	Necrotic	0.064 ± 0.004 (0.001)	1.476 ± 0.058 (0.017)	0.841 ± 0.114 (0.034)	8.947 ± 0.688 (0.207)	0.126 ± 0.004 (0.001)	0.05 ± 0.001* (0.0003)
ON	Sclerotic	0.061 ± 0.004* (0.001)	1.464 ± 0.066 (0.020)	0.869 ± 0.127 (0.038)	9.018 ± 0.568 (0.171)	0.127 ± 0.005 (0.002)	0.049 ± 0.001* (0.0003)
ON	Distant	0.061 ± 0.004 (0.001)	1.492 ± 0.072 (0.022)	0.841 ± 0.121 (0.036)	8.913 ± 0.537 (0.162)	0.126 ± 0.004 (0.001)	0.049 ± 0.001* (0.0003)
Control	Necrotic equivalent	0.075 ± 0.016 (0.005)	1.505 ± 0.069 (0.021)	0.763 ± 0.152 (0.046)	9.624 ± 0.716 (0.216)	0.126 ± 0.004 (0.001)	0.051 ± 0.001 (0.0003)
Control	Sclerotic equivalent	0.074 ± 0.015 (0.005)	1.453 ± 0.066 (0.02)	0.749 ± 0.136 (0.041)	9.722 ± 0.640 (0.193)	0.127 ± 0.003 (0.0009)	0.051 ± 0.002 (0.0006)
Control	Distant equivalent	0.075 ± 0.015 (0.005)	1.465 ± 0.104 (0.031)	0.752 ± 0.115 (0.035)	9.411 ± 0.353 (0.106)	0.127 ± 0.004 (0.001)	0.051 ± 0.002 (0.0006)

None of these differences were significant between zones within groups. *Corrected p-value < 0.05 (univariate between group differences, none of these differences were significant after multivariate analysis).

#### Inside-group comparisons

The PCPs were not significantly different between the three zones in the control group. The PCPs were not significantly different between the necrotic, sclerotic and distant zones in the ON group.

#### Between-group comparisons

##### Uni and bi-variate analysis.

 No significant differences were found regarding the mineral-to-matrix ratio, carbonate B-substitution, collagen maturity and the hydroxyproline-to-proline ratio for any of the matched zones. Crystallinity was significantly decreased in all zones of the ON group compared with the control group (p = 0.04). The relative proteoglycan content was significantly decreased in the sclerotic zone of ON patients compared with the matched zone of controls (p = 0.03). This decrease was not significant in the necrotic zone (p = 0.28) and tended to be significant in the distant zone (p = 0.07).

##### Multivariate analysis.

 Parameters with significant differences in bivariate analysis were tested for multivariate analysis with adjustment on age and gender using a mixed linear model.

**Figure 2:**
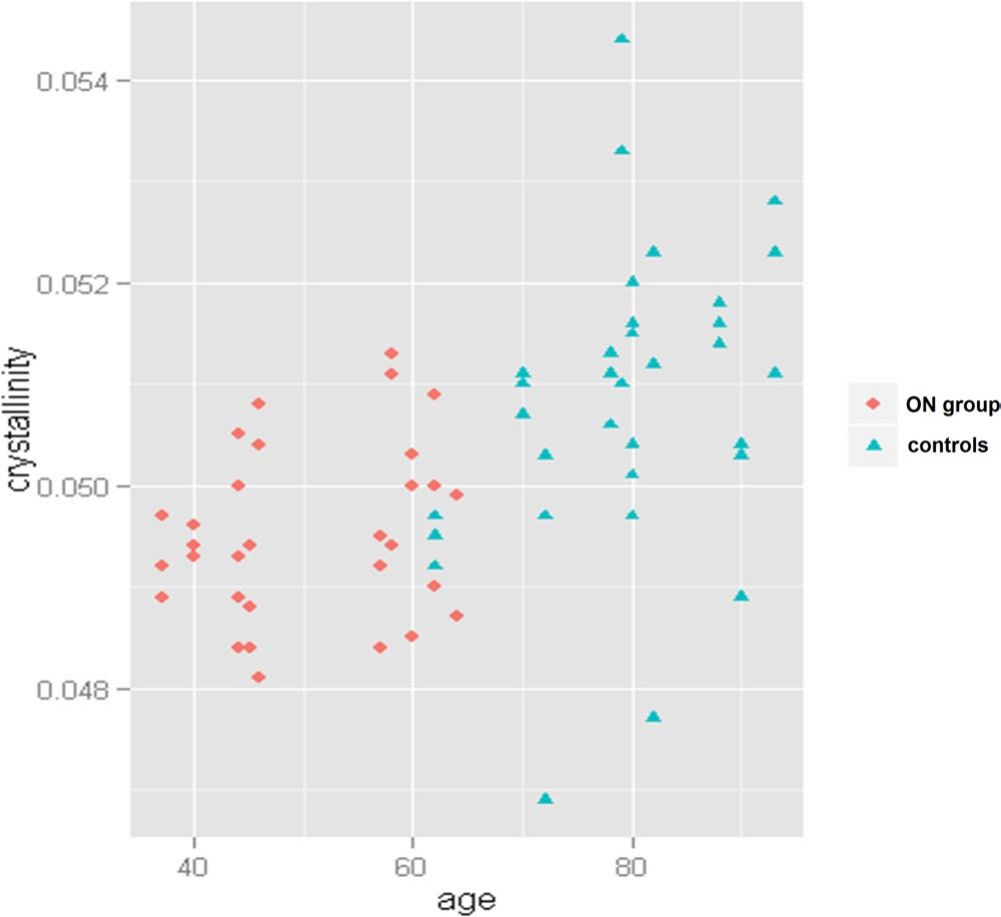
Age-related distribution of crystallinity values.

The model regarding crystallinity was found valid after descending selection. Selected variables were age (p = 0.026) and gender (p = 0.056) suggesting that observed differences of crystallinity between groups were age-related (Fig. 2).

The residual normality of the model for the proteoglycan content was negative even after log transformation of the variable. Non-linear models were considered but not performed due to a risk of misinterpreting the data. The decrease of the proteoglycan relative content in bivariate analysis was not explained by multivariate analysis. However, in order to measure a suspected influence of age on the relative proteoglycan content in the intermediary zone, a Spearman correlation coefficient was calculated. The coefficient was statistically significant (0.57, p = 0.013) showing that the relative proteoglycan content was positively correlated with age.

### Histology

**Figure 3:**
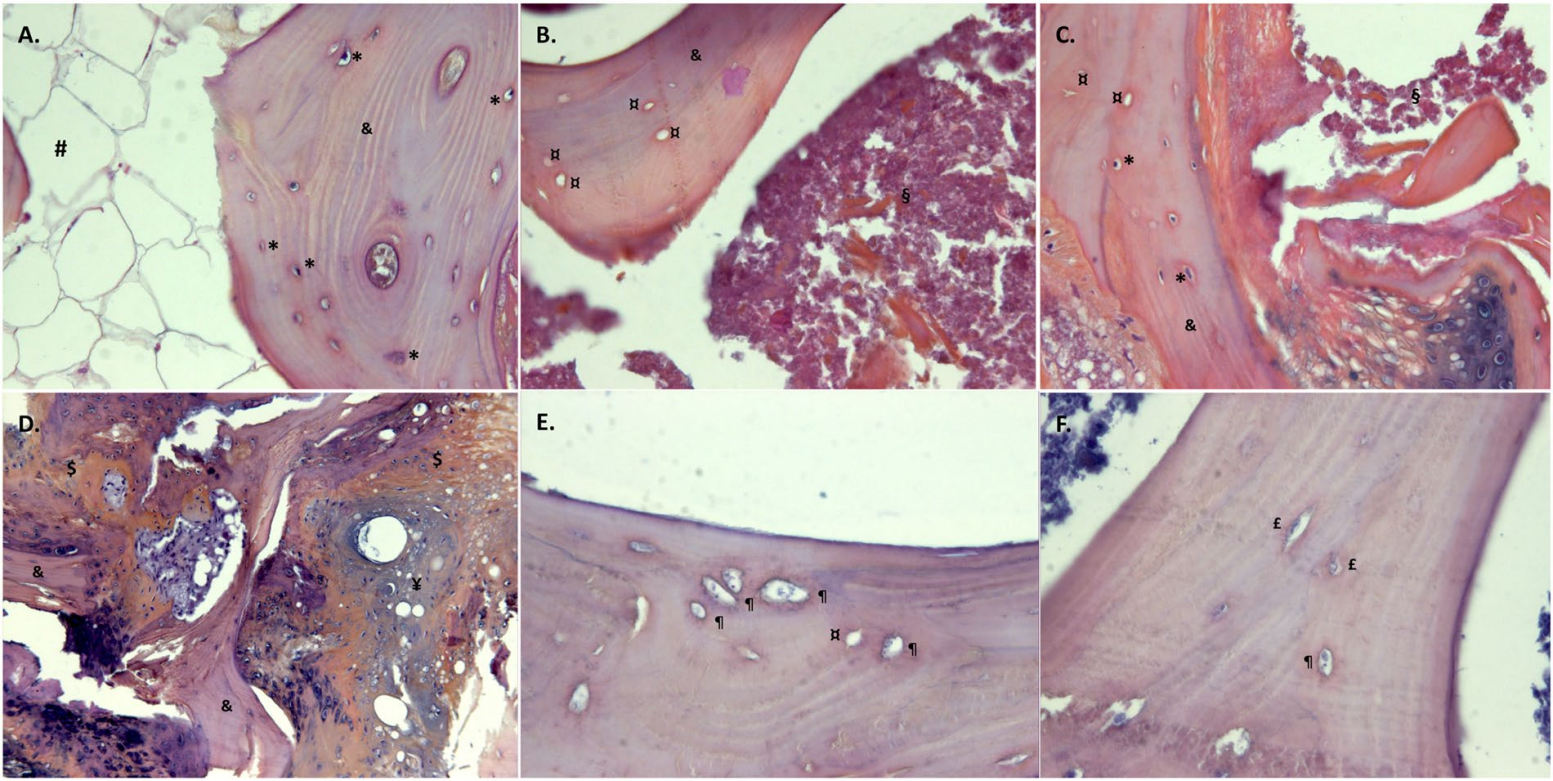
Histological examination using hemtoxylin-eosin-saffron staining. (**A**) Control sclerotic-equivalent zone (x200); (**B**). Necrotic zone (ON group) (x200); (**C**). Sclerotic zone (ON group) (x200); (**D**). Sclerotic zone with coexisting normal trabeculae, woven and chondroid bone (ON group) (x200); (**E**). pyknotic apoptotic osteocytes (ON group) (x400); (**F**). coexistence of pyknotic osteocytes others presenting with nuclei showing spread and faded chromatin (x400). ^&^Normal trabecula, ^$^woven bone, ^¥^chondroid bone, ^#^normal medullary space, ^§^necrotic medullary space, *intact osteocyte, ^¶^apoptotic osteocyte, ¤empty osteocyte lacuna. ON: osteonecrosis.

Histological analysis confirmed the diagnosis of ON with extended bone marrow necrosis in the necrotic zone. The necrosis was extended to the sclerotic zone in some cases. The bone marrow was normal in the distant zone. Osteocyte lacunae were either empty or filled with dead cells in the necrotic zone. Some dead or intact osteocytes were filling most osteocytes lacunae in the sclerotic zone, with some of them remaining empty. Almost exclusively intact osteocytes were found in the distant zone. Osteocyte lacunae filled with pyknotic cellular structures coexisted with others presenting with nuclei showing spread and faded chromatin. In the sclerotic zone could be found new bone structure due to repairing processes with chondroid and woven bone associated with normal trabeculae (Fig. 3).

Examination of the three zones from the control group showed normal bone structure with intact osteocytes.

## Discussion

This study provides evidence that the molecular composition and structure of the trabecular bone is not modified during end-stage non-traumatic ON of the femoral head despite extensive cell death.

The mineral and organic compositions of bone were generally not modified across zones and between groups. These results are in agreement with a previous study of piglets using microscale techniques^25^. Indeed, using X-ray absorbance near edge structure and Raman spectroscopy, molecular composition was found to be unchanged in the necrotic bone apart from a higher carbonate substitution in the necrotic zone. This increased carbonate substitution in the necrotic zone, found only in the animal model, could be a result of early stage processes, differences related to animal pathophysiology, but also a result of sample fixation that could have affected carbonate substitution^24^. Aruwajoye *et al.* found that molecular stability of the necrotic zone was not echoed by structural stability as architectural trabecular modifications were seen in the necrotic zone both with scanning electron microscopy and micro-computed tomography^25^. Wang *et al.* tested the biomechanical properties of human necrotic femoral heads using nano-indentation. Once again, bone nanoscopic biomechanical properties were similar between necrotic and healthy regions despite clear architectural alterations of the necrotic bone evaluated by micro-computed tomography^17^.

The decrease of the relative proteoglycan content in the sclerotic region is probably age-related and linked to increased bone remodelling in this zone. Proteoglycan content was previously shown to increase with age^27^ as further confirmed by a significant correlation between the two variables in our study. In addition, it is also known that proteoglycans act as a scaffold of bone mineralisation and down-regulate the bone mineral apposition rate to obtain a well-organized bone^27, 28^. Histological examination showed the presence of woven bone that corresponds to rapid, disorganised and unregulated new bone apposition expected to have low proteoglycan content.

The absence of modification of bone composition despite extensive osteocyte death suggests that osteocytes may not play a central role in ON. Previous studies found an increased cell apoptosis rate in the osteocyte/osteoblast lineage and hypothesized that osteocyte apoptosis in the cancellous bone was a key mechanism in the pathogenesis of osteonecrosis^12, 15, 29–31^. Osteocytes are known to act on the mineral homeostasis of bone^32^. There is however no evidence from our study that the mineral composition is modified, advocating that the observed osteocyte death did not impact on the trabecular bone quality. This would imply that osteocyte death may simply be a marker of trabecular bone stress rather than an active participant to its collapse. Consistently with this hypothesis, Weinstein *et al.* have shown that this cell death is predominant near the fractured subchondral region, suggesting that trabecular bone suffers from the nearby defective subchondral bone^29^. Mutijima *et al.* also showed an increase of apoptotic osteocytes in the sclerotic region^15^ that is also in contact with the compromised necrotic zone^17^.

Our study supports the hypothesis that the quality of trabecular bone may not be affected directly by ON. Several studies looked into potential modifications of the trabecular architecture of human or animal femoral heads undergoing or having undergone osteonecrosis. Two studies compared the remaining “healthy” trabecular bone using micro-computed tomography of human osteonecrotic femoral heads versus osteoarthritic controls and none of them found differences^33, 34^. The same results were found in a model of corticosteroid-induced ON of the femoral head in bipedal emus^35^. The total preservation of the trabecular microarchitecture, apart from the necrotic zone, is intriguing. Indeed, if trabecular microarchitectural modifications were a pathophysiological feature of the disease, it can be hypothesized that they would not be cut off-limited to the collapsing necrotic zone but would also be present to some extent in its periphery. The microarchitectural modifications observed only in the necrotic zone could rather be the consequence of excessive mechanical stress and aggravated by increased bone resorption^36^. The study of osteoclast activity and growth factor expression in human ON showed a particular increase in the sclerotic trabecular region and also nearby subchondral fractures suggesting that these mechanisms react to mechanically compromised zones^37^.

We acknowledge that this study has limitations. The sample size of the study is modest but keeping in the context of non-traumatic ON, contrary to many studies^17, 25, 36, 37^, a control group was provided and the population size of the control group was larger than observed in most similar studies^15, 33, 35^. Age-related increase of crystallinity (and to some extent the decrease of the proteoglycan content) was an expected between-group difference^38^ and was confirmed by multivariate analysis. Age differences between groups did not seem to have induced significant differences regarding the other parameters according to univariate analysis. The histological analyses that were performed with hematoxylin-erythrosin-saffron (HES) staining are qualitative only and no TUNEL assay was performed, therefore these results must be interpreted with caution. Nonetheless, given the specific morphological aspects of the various cell deaths^39^, these first observations of both necrotic and apoptotic osteocytes are convincing.

Together with biomechanical and microarchitectural studies, our study advocates for a relative innocence of cancellous bone in both the disease pathogenesis and the collapse of the osteonecrotic femoral head. In the meantime, evidence is being gathered against suspect number two: subchondral bone. Osteoclast activity and growth factors tend to be concentrated in the subchondral region^17, 37^. Microarchitectural modifications of the subchondral bone and especially thinning and increased porosity of the subchondral plate have been observed in animal models^35, 40^. In addition, clinical outcomes of hip resurfacing preserving the trabecular bone but replacing the subchondral bone are very promising even in case of extensive osteonecrosis^41–44^. Further controlled studies on human ON exploring modifications of the subchondral bone involving its architecture, composition and structure within the necrotic zone but also in more distant regions are required to provide a better understanding of the pathophysiology and course of ON.

## Methods

### Samples

**Figure 4:**
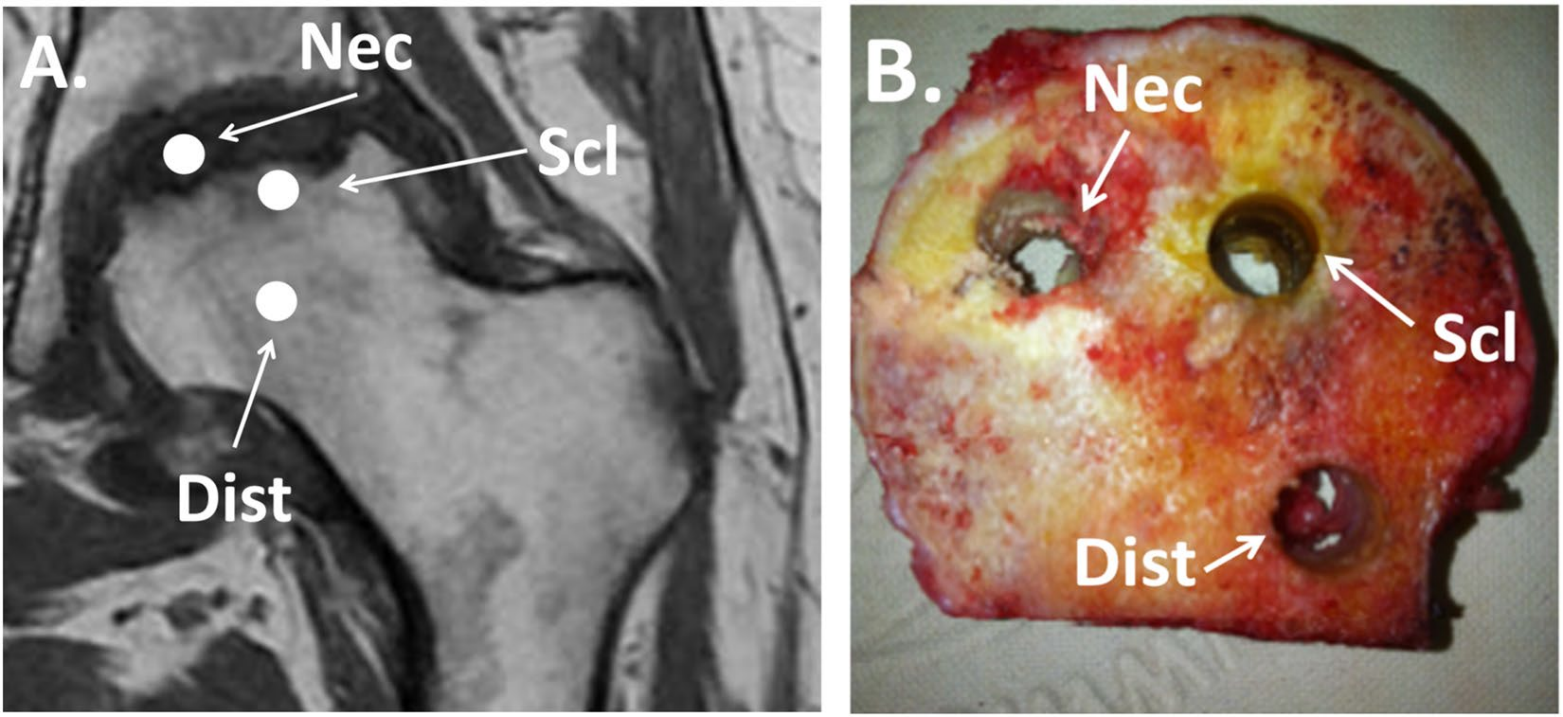
Magnetic resonance imaging (**A**) and macroscopic (**B**) localizations of the three sampled zones. Nec = necrotic zone, Scl = sclerotic zone, Dist = distant zone.

Between April 2010 and March 2014, samples from femoral heads were harvested from eleven patients and eleven cadaveric controls. The patients gave informed consent to participate and the study was approved by the ethical review board of the *Direction Générale de la recherche et de l’innovation* (French Ministry for Research) (DC-2008-642). The study was carried out in accordance with the World Medical Association Declaration of Helsinki. Patients undergoing total hip arthroplasty with the diagnosis of magnetic resonance imaging (MRI)-proven non-traumatic ON of the femoral head were included in the study. All patients were male (mean age (±SD) 50.6 (±9.6) years). Demographic and clinical characteristics of patients are detailed in Table 2. The ON group was composed of biopsies of the femoral heads collected in the operating room just after the resection of the femoral head. Biopsies were performed using a 10 mm diameter and 25 mm hole saw in three zones pre-determined by a systematically performed pre-operative MRI examination and confirmed by visual macroscopic observation: the necrotic zone, the sclerotic zone and in the distant zone (far off the necrotic zone) (Fig. 4).

**Table 2:** Disease history and patients’ characteristics.

Patient number	Age (y.o)	Gender	Side	Bilateral osteonecrosis	Disease duration (months)	Cause	FICAT stage
1	58	male	Left	Unknown	36	Alcohol abuse	4
2	45	male	Left	Yes	6	Alcohol abuse	3
3	46	male	Right	Yes	24	Alcohol abuse	3
4	64	male	Right	Yes	12	Glucocorticoids use	4
5	57	male	Right	Yes	unknown	Alcohol abuse	3
6	44	male	Left	Yes	unknown	Alcohol abuse	4
7	40	male	Right	Yes	36	Alcohol abuse	4
8	62	male	Left	Yes	unknown	Alcohol abuse	4
9	44	male	Right	Unknown	unknown	Alcohol abuse	4
10	60	male	Right	Yes	unknown	Alcohol abuse	4
11	37	male	Right	Yes	18	Alcohol abuse	3

The control group was composed of bone biopsies from cadaveric donors provided by the Lille University anatomy department. The donors had informed of their will to donate their body to science after their death. The donors had no recorded history of bone disease. Femoral heads were harvested immediately upon arrival of the corpses at the anatomy department before any embalmment procedure was performed^24^. Samples were harvested from the corpses of seven male and four female subjects who died at a mean age of 79.4 (±9.1) years. The femur was sectioned at the base of the neck to harvest the entire femoral head from each subject. Three separate biopsies were sampled in equivalent zones comparatively to the ON group: below the subchondral region, base of the femoral neck, and in between the two.

### Sample preparation

Samples were stored at −80 °C until analysis. An 8 mm height section of each biopsy was cut 8 mm below the cartilage surface using a speed diamond blade saw. The section was fixed in 70% ethanol solution for 48 H to avoid sample degradation during the Raman analysis, then polished using abrasive papers with decreasing grain size (30, 3 and 0.3 µm) and set on a microscope slide. A contiguous sample of the same size was used for histology.

### Histology

The samples were decalcified with a solution of formaldehyde, methanol and formic acid. Sections of paraffin-embedded sample blocks were stained with HES. Optical microscopic examination was performed. The extents of necrosis and osteocyte viability were assessed by qualitative histological examination across the three zones.

### Raman Acquisitions

**Figure 5:**
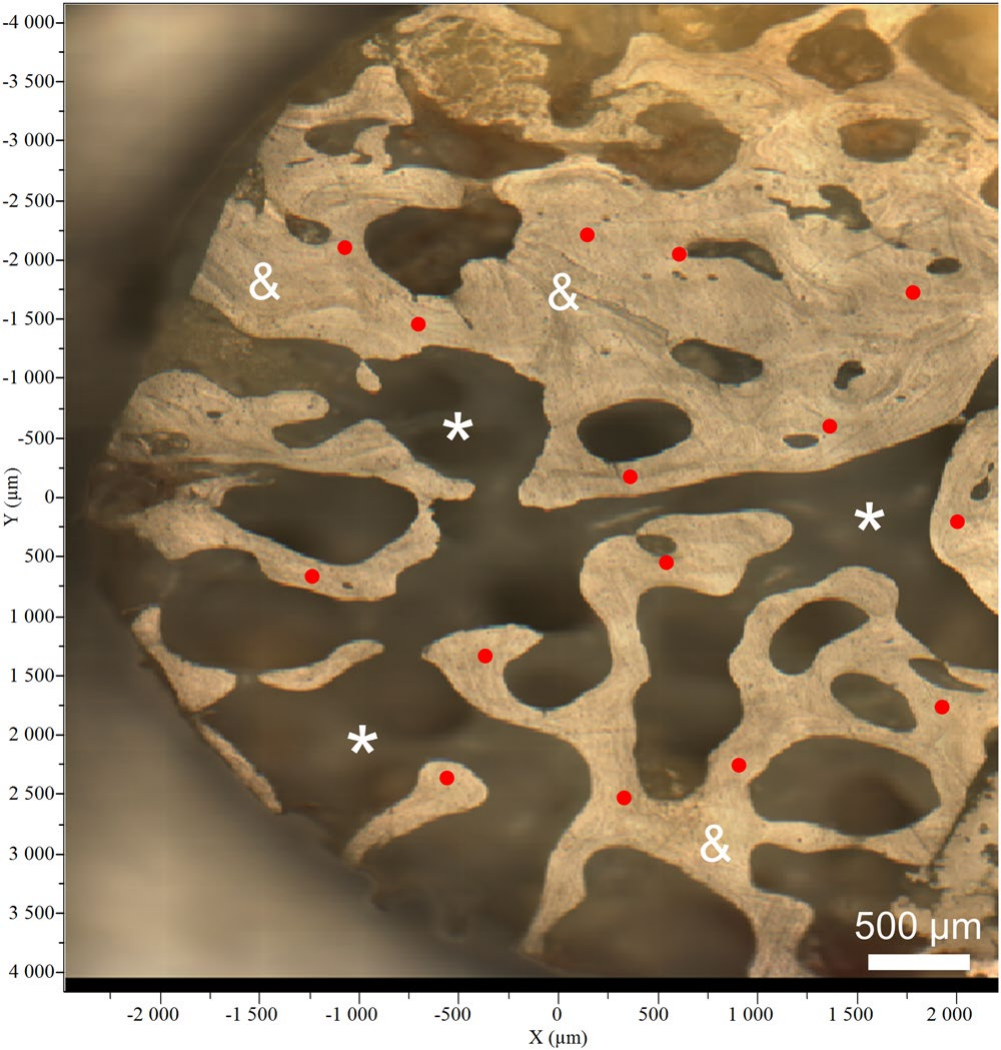
Partial overview of a single sample in confocal microscopy (X10) with each of the 15 (/30 of the entire sample) selected spots selected for Raman spectral acquisition (red dots). *Marrow regions, ^&^mineral trabeculae.

Once the sample is grinded to obtain an even surface, mineralized trabeculae are distinguished from bone marrow regions, and zones of the trabeculae were pre-selected for Raman spectral acquisitions (Fig. 5). Spectra were acquired with a Raman microspectrometer LabRAM HR800 (HORIBA, Jobin-Yvon, France) provided with DuoScan technology. The instrument is equipped with a XYZ motorized stage and a diode laser at 785 nm. DuoScan technology provides an average spectrum representative of a rastered area giving the opportunity to explore more rapidly broader samples^45^. The DuoScan averaging mode was used with an ×50 objective and a rastered area of 30 × 30 µm. Thirty spectra were acquired for each sample, with an integration time of 60 seconds and 5 accumulations. The spectral range was 300 to 1700 cm^−1^ with a resolution of 4 cm^−1^.

All Raman spectra were processed using Labspec software (HORIBA, Jobin-Yvon, France). A Savitzky-Golay smoothing filter (filter width: 3; polynomial order: 2) and polynomial baseline correction (degree 4) were applied to the data set prior to evaluation of physicochemical parameters.

### Bone physicochemical parameters

The physicochemical parameters characterizing bone composition and structure have been described previously^18^. Mineral-to-matrix ratio was calculated from the ratio of the phosphate symmetric stretch band intensity (960 cm^−1^) to the intensity of vibrations resulting primarily from the CH_2_ side-chains of collagen molecules (1450 cm^−1^). This parameter measures relative bone mineral-to-organic content that provides an assessment of mineralization. Crystallinity was calculated as the inverse of the full width at half maximum (FWHM) of the phosphate symmetric stretch band. Crystallinity gives an assessment of crystal size and perfection. Type-B carbonatation was calculated as the ratio between the respective intensity of the type-B carbonate symmetric stretch band (at 1073 cm^−1^) and the phosphate symmetric stretch band. This ratio represents the level of type-B carbonate substitution in the apatite crystal. The relative proteoglycan content was calculated from the ratio of integrated areas of the glycosaminoglycan (GAG)/CH_3_ band (1365–1390 cm^−1^) to the amide III band (1243–1269 cm^−1^). The hydroxyproline-to-proline ratio was calculated as the intensity ratio of hydroxyproline (877 cm^−1^) and proline (853 cm^−1^). The study of the hydroxyproline and proline Raman bands was shown to give a reliable evaluation of post-translational modifications of collagen in bone^22, 46, 47^. This ratio provides an assessment of the collagen secondary structure with implications for bone mineralization capability^48^. Collagen maturity was calculated as the intensity ratio of two sub-component bands in the amide I band commonly described approximately at 1660 cm^−1^ for the amide I peak and 1690 cm^−1^ for the subsequent shoulder^22^.

### Statistical analysis

Statistical analysis was performed using the R software. The value of each physicochemical parameter of each biopsy sample was the average of the 30 spectra collected. Results are presented as mean (±SD (SE)). Comparisons between the three zones inside groups were performed using the non-parametric Quade test as repeated measures within the same subjects were considered. The Mann-Whitney-Wilcoxon test was used to compare matched zones of the ON and control groups. P-values were corrected for each value using Holm’s technique to avoid the inflation of the α risk due to repeated comparisons of each zone. Multivariate analysis was performed for parameters with significant p-value in bi-variate analysis using mixed linear models given the repeated measurements (3 zones) performed on each subject. Age, sex, group, and zone were integrated as fixed effects in the models. The small sample size required the application of a variable selection procedure. The step by step, backward method, based on the Akaïke criterion was chosen. The model validation was assessed graphically using the residuals (normality and homoscedasticity). Statistical significance was assigned to p < 0.05.
